# Inhibition of Tumor Growth of Human Hepatocellular Carcinoma HepG2 Cells in a Nude Mouse Xenograft Model by the Total Flavonoids from* Arachniodes exilis*

**DOI:** 10.1155/2017/5310563

**Published:** 2017-11-19

**Authors:** Huimin Li, Dengzhao Jiang, Lei Zhang, Jiazhong Wu

**Affiliations:** ^1^School of Basic Medical Science, Jiujiang University, No. 320 Xunyang Eastern Road, Jiujiang, Jiangxi 332000, China; ^2^Key Laboratory of System Biomedicine of Jiangxi Province, Jiujiang, Jiangxi 332000, China; ^3^School of Pharmacy and Life Sciences, Jiujiang University, No. 320 Xunyang Eastern Road, Jiujiang, Jiangxi 332000, China

## Abstract

A tumor growth model of human hepatocellular carcinoma HepG2 cells in nude mice was employed to investigate the antitumor activity of the total flavonoids extracted from* Arachniodes exilis* (TFAE)* in vivo*. Several biochemical assays including hematoxylin-eosin (HE) staining, immunohistochemistry, and Western blot were performed to elucidate the mechanism of action of total flavonoids extracted from* Arachniodes exilis* (TFAE). The results showed that TFAE effectively inhibited the tumor growth of hepatocellular carcinoma in nude mice and had no significant effect on body weight, blood system, and functions of liver and kidney. Expression levels of proapoptotic proteins Bax and cleaved caspase-3 remarkably increased while the expressions of Bcl-2, HIF-1*α*, and VEGF were suppressed by TFAE. These results suggested that the antitumor potential of TFEA was implied by the apoptosis of tumor cells and the inhibition of angiogenesis in tumor tissue.

## 1. Introduction

Hepatocellular carcinoma (HCC) is the fifth common form of liver cancer worldwide, especially in Asia and Africa [1]. Surgical resection is the most efficient therapeutic strategy, but only 20% of patients are able to receive surgical therapy [2]. Currently some obvious problems exist in clinical therapy of HCC, such as early diagnosis, high recurrence rate, and lack of specific treatment and innovative medicines [3]. Many studies have shown that traditional Chinese medicines (TCM) may be able to retard HCC progression with multiple actions, either alone or in combination with other conventional therapies to improve life quality of HCC patients [4–6]. Moreover, TCM (including plants, animal parts, and minerals) have drawn a great deal of attention in recent years for their potential in the treatment of HCC [7].

*Arachniodes exilis (A. exilis)*, a fern belonging to* Arachniodes* species (Dryopteridaceae), is widely distributed in the tropical and subtropical moist areas of the world, especially in the eastern and southeastern Asia. In China, this plant is mainly distributed in the south areas of the Yangtze River and Shandong and Henan provinces.* A. exilis* has been used as a folk medicine for a long time to treat acute jaundice hepatitis, arthritis, lumbago, dysentery, and burn injuries and proved to exhibit antibacterial, anti-inflammatory, and sedative activities by modern pharmacological studies [8]. Studies indicate that Dryopteridaceae plants usually possess antivirus and anticancer effect, which is based on the bioactive constituents of flavonoids and phloroglucinol derivatives [9]. Mechanisms of anticancer effect of Dryopteridaceae plants are different from those of the common chemotherapeutic drugs. They possess the cytotoxicity towards tumor cells, meanwhile, without damage to the hematopoietic stem cells [10].

Our previous research suggested that TFAE was able to induce apoptosis of HepG2 cells* in vitro* [11]. In this study, the nude mice xenograft model was employed to investigate the mechanisms of antitumor effect of TFAE, such as the inhibition rate, biochemical index, HE staining, immunohistochemistry, and Western blot.

## 2. Materials and Methods

### 2.1. Materials

The total flavonoids from* Arachniodes exilis* (TFAE) were extracted and purified in our laboratory. First, appropriate dried powder (20–30 mesh) of* A. Exilis* roots was extracted by using 60% ethanol (solid-liquid ratio of 1 : 30) with ultrasonication for 3 times, each time for 2 hours. The extract of* A. exilis* was obtained by concentrating the extracting solution to dryness in a rotary evaporator and a freeze dryer; then the extract was purified by the polyamide column chromatography with 70% ethanol to get TFAE. The total flavonoids in TFAE were estimated as rutin equivalent and the content was measured on the basis of the rutin calibration curve (*Y* = 82.645*X* + 2.661 *R*^2^ = 0.9998, where *Y* is the absorbance and *X* is the concentration (*μ*g/ml)) [11]. The content of the total flavonoids in TFAE was 82.86% after the polyamide purification process.

The DMEM/F-12 medium was from HyClone (Thermo Fisher Scientific, Massachusetts, USA) and fetal bovine serum was from Trans Gen Biotech Biotechnologies Co., Ltd (Beijing, China). The trypsin, DMSO, and agarose were all from Solarbio Technology Co., Ltd (Beijing, China). The total protein extraction kit was from Aidlab Biotechnologies Co., Ltd (Beijing, China). The 4% paraformaldehyde fixative solution and HE staining solution were from Biossic Biotechnology Co., Ltd (Wuhan, China). The Bcl-2, Bax, VEGF, and HIF-1*α* immunohistochemistry kit were from eBioscience (Thermo Fisher Scientific, Massachusetts, USA). The Bax, HIF-1*α*, and VEGF antibodies were from Santa Cruz Biotechnology, Inc. (California, USA), and the Bcl-2, cleaved caspase-3, and *β*-actin antibodies were obtained from Abcam (Cambridge, UK). The PVDF membrane was from Millipore. Additional reagents and solvents used in this study were commercial products of analytical grade.

### 2.2. Methods

Human hepatoma HepG2 cells were inoculated into immunodeficient nude mice to establish the animal model of liver tumor. Several indicators related to tumor growth were evaluated. The experimental mice were also investigated by biochemical and histological detection. Furthermore, the expressions of apoptosis-related proteins in tumor tissues were determined to explore the underlying mechanisms for the antitumor activity of TFAE.

#### 2.2.1. Establishment of the Tumor Models in Nude Mice

Male BALB/c-nu mice of SPF grade, 4-5 week-old, were purchased from Hunan SJA Laboratory Animal Co., Ltd, in Changsha, China [License Number SCXK (Xiang) 2014-0002]. The mice were bred in animal house (SPF degree) with barrier system assisted with apinoid laminar flow chamber in the Experimental Animal Center of Huazhong University of Science and Technology. These animals were housed under controlled conditions (temperature 22 ± 2°C, relative humidity 50 ± 10%) with a natural light-dark cycle for one week before the experiment was carried out. The animal studies were conducted in accordance with the Provision and General Recommendation of Chinese Experimental Animals Administration Legislation. The HepG2 cells of logarithmic phase were washed twice by serum-free culture solution and resuspended to a concentration of about 1 × 10^7^/ml. The cells (0.2 ml) were inoculated subcutaneously on the back of each nude mouse.

#### 2.2.2. The Administration Groups of Nude Mice

After the inoculation, the animals were randomly divided into four groups: the negative control group, the positive control group, the TFAE low-dose group, and the TFAE high-dose group. The administration was designed according to Table 1. The drug for the negative control group was prepared as follows: 1.0 g of sodium carboxymethylcellulose (CMC-Na) and 200 ml of distilled water were fully swollen and dissolved to yield a 0.5% CMC-Na solution. 5-FU was used as the drug for the positive control group and dissolved with distilled water to prepare the administrative solution. The TFAE was dissolved in the 0.5% CMC-Na solution to obtain the sample solution. All animals were given once intragastric administration (ig) every other day which lasted for 4 weeks. The mice were weighed and the tumor growth in nude mice was monitored carefully once a week.

#### 2.2.3. Growth Indicators of Tumors in Mice

The long diameter (*a*), short diameter (*b*), and volume (*V*, mm^2^) of tumors in the mice were measured; relative tumor volume (RTV) and the relative rate of tumor growth rate *T*/*C* (%) were calculated.

(1) V=a×b22,RTV=VtV0.

*V*_0_ is the volume measured after the first administration; *Vt* is the volume measured after each administration.

(2) $$\begin{array}{c} \frac{T}{C}\left(\;\%\;\right)=\frac{T_{RTV}}{C_{RTV}}. \end{array}$$

*T*_RTV_ is the RTV of mice in positive control group and TFAE administration groups; *C*_RTV_ is the RTV of mice in the control group.

#### 2.2.4. Pathological Examination

After 24 h of the last administration, mice were sacrificed under anaesthesia and dissected. Blood samples and tumor tissues were collected. A part of the tumor tissue was fixed by 4% paraformaldehyde for pathology detection, and the remaining part was frozen in refrigerator.

*(1) Biological Assays*. The blood analysis was performed using the whole blood samples to measure the number of red blood cells (RBC), white blood cells (WBC), blood platelet (PLT), and hemoglobin (HB) by using DxH800 automatic hematology analyzer (BECKMAN COULTER). Alanine aminotransferase (ALT), aspartate transaminase (AST), blood urea nitrogen (BUN), and creatinine (Cr) were measured by AU5821 automatic biochemical analyzer (BECKMAN COULTER). All of the work had been done in the hospital attached to the Tongji Medical College, Huazhong Science and Technology University.

*(2) Morphological Study of the Tumor Tissues. *Tumor tissue was stained by hematoxylin-eosin (HE) and observed under a microscope.

*(3) Immunohistochemistry Analysis*. The slice of tumor tissue was prepared by conventional process. Immunohistochemical analysis of Bcl-2, Bax, HIF-1*α*, and VEGF expressions in tumor tissue was applied according the guideline of each test kit. The following antibodies were used: Bcl-2 (ab32124, Abcam, Cambridge, UK; 1 : 100), Bax (Sc-526, Santa Cruz, California, USA; 1 : 100), HIF-1*α* (Sc-13515, Santa Cruz; 1 : 100) and VEGF (SC-57496, Santa Cruz; 1 : 100).

*(4) Western Blot Assay*. Western blot assay was conducted to detect expression levels of Bax, Bcl-2, cleaved caspase-3, HIF-1*α*, and VEGF. Total proteins of the tumor tissues were extracted using a total protein assay kit. After the determination of protein contents via the Bio-Rad protein assay, these protein samples (40–50 *μ*g) were separated by 12% SDS-PAGE and electrotransferred to nitrocellulose membrane. The membranes were washed in Tris-buffered saline containing 0.1% Tween 20 (TBST), blocked with 5% nonfat milk in TBST for 1 h at room temperature, and incubated with following specific antibodies: Bax (Sc-526, Santa Cruz, California, USA; 1 : 1000), HIF-1*α* (Sc-13515, Santa Cruz; 1 : 800), VEGF (SC-57496, Santa Cruz; 1 : 1000), Bcl-2 (ab32124, Abcam, Cambridge, UK; 1 : 1000), cleaved caspase-3 (ab2302, Abcam; 1 : 1000), and *β*-actin (ab115777, Abcam; 1 : 1000) overnight at 4°C. The membranes were washed three times in TBST, followed by incubation with the appropriate horseradish peroxidase- (HRP-) linked secondary antibodies (Proteintech, Wuhan, China; 1 : 5000) for 1 h at room temperature. The specific proteins on the blots were developed using enhanced chemiluminescence (ECL; Vazyme Biotech Co., Ltd., Nanjing, China) and visualized as bands on CL-XPosure film (Thermo Fisher Scientific, Inc.). The optical densities of the bands were measured on the GS710 Densitometer and analyzed using Quantity One image analysis software version 4.6 (Bio-Rad Laboratories, Inc.).

#### 2.2.5. Statistical Analysis

Data were expressed as mean ± standard deviation (SD). All data were analyzed using one-way analysis of variance (ANOVA), followed by Dunnett's test for pairwise comparison. A *p* value of <0.05 was considered as an indication for statistical significance. The SPSS 19.0 software (IBM Corporation, Armonk, NY, USA) was used to analyze the data.

## 3. Results

### 3.1. Observation of the Animals during the Experiment

After a certain period of inoculation with HepG2 cells, obvious lumps were visible under the skin at the inoculation site, indicating the successful establishment of heterotopic transplantation model of human hepatoma HepG2 cells. Animals in each group behaved normally in eating, drinking, and excretion during the experiment. After the experiment, nude mice of each group were dissected, and no significant organ damage was observed.

The body weight of animals in each group increased as shown in Table 2. Statistical analysis showed that there was no significant difference between the average weights of animals in each group, suggesting that the TFAE had no effect on body weight of the hepatocellular carcinoma-bearing nude mice.

### 3.2. Effect of TFAE on the Tumor Growth in Nude Mice

**Figure 1:**
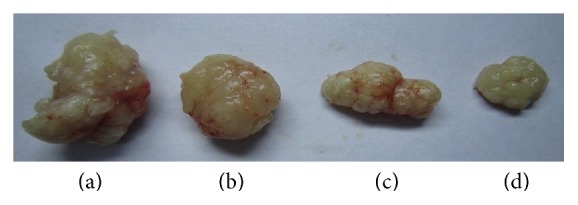
Effect of TFAE on the tumor growth in nude mice. (a) Negative control group; (b) TFAE low-dose group; (c) TFAE high-dose group; (d) positive control group.

As shown in Figure 1, the animals treated with 5-FU and TFAE showed significantly lower tumor growth rate than the mice in negative control group, revealing that administration with TFAE effectively inhibited the tumor growth in the nude mice.

**Figure 2:**
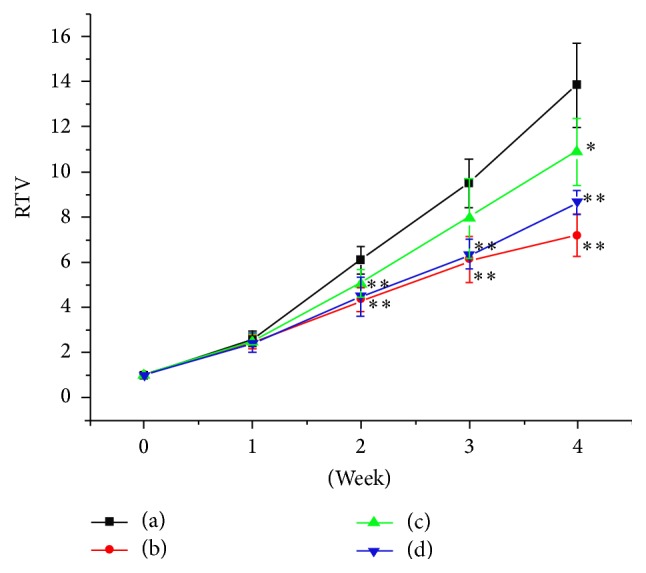
Changes in the relative tumor volume of tumor-bearing mice in each group. (a) Negative control group; (b) positive control group; (c) TFAE low-dose group; (d) TFAE high-dose group; ^*∗*^*p* < 0.05 and ^*∗∗*^*p* < 0.01, compared with the negative control.

Changes on the relative tumor volume of mice in every experimental group were analyzed and shown in Figure 2.

### 3.3. The Impact of TFAE on the Blood System and Functions of Liver and Kidney of the HCC Animal Model

Results are listed in Table 3. WBC in the positive control group (5-FU treatment group) were significantly lower than those in the negative control group while the content of ALT, AST, and BUN was significantly higher on the contrary (*p* < 0.05). It was shown that mice in the positive control group (5-FU treatment group) showed significant differences in the indicators of blood system as well as liver and kidney functions when compared with animals in the negative control group, proving that 5-FU had toxicity to the blood system, liver, and kidney. Meanwhile, the results also suggested that mice in TFAE administration groups had no significant difference in these indicators compared with mice in negative control group. This indicated that TFAE have no obvious damage on the blood system, liver, and kidney.

### 3.4. Morphology of Tumor Tissue

**Figure 3:**
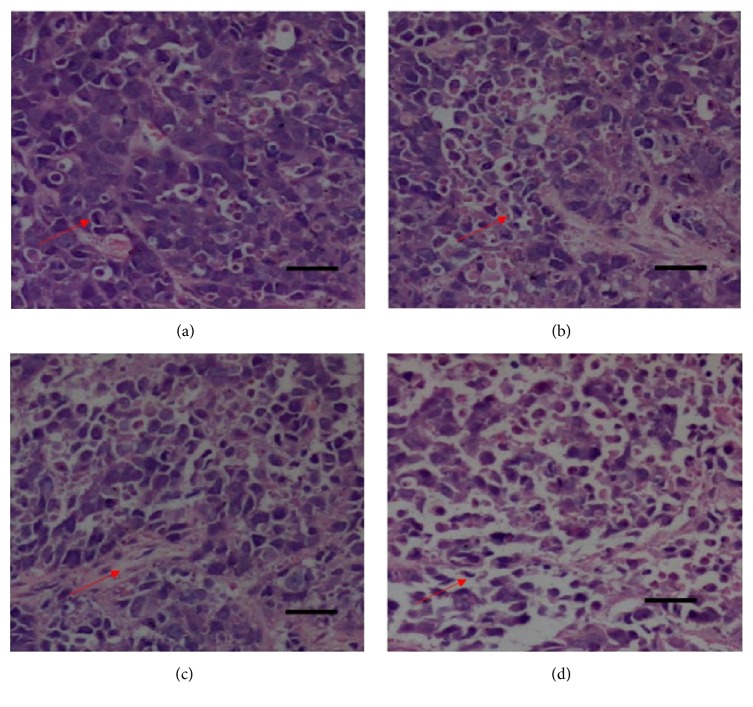
HE staining of tumor tissues belonged to mice of different administration groups (×100). (a) Negative control group; (b) TFAE low-dose group; (c) TFAE high-dose group; (d) positive control group.

HE stained samples of tumor tissues of tumor-bearing mice were shown in Figure 3. The tumor cells in the tissues of nude mice belonging to negative control group were closely aligned and possessed bigger cell nucleus and higher nuclear/cytoplasm ratio than those in the other three groups. After the treatment with TFAE or positive control drug 5-FU, however, cancer cells exhibited phenomena such as sparse arrangement, cell shrinkage, fragmentation, and chromatin disappearance, indicating necrosis at varying degrees happened in the cancer cells.

### 3.5. Immunohistochemical Assay

**Figure 4:**
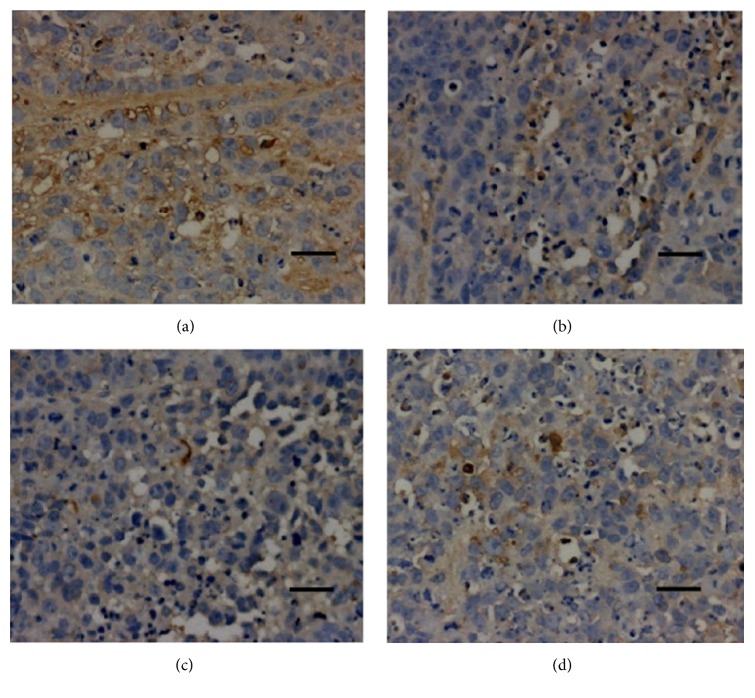
Expression of apoptosis-related protein Bcl-2 in tumor tissues (×100). (a) Negative control group; (b) TFAE low-dose group; (c) TFAE high-dose group; (d) positive control group.

Immunohistochemical tests were applied to detect the expressions of Bcl-2, Bax, HIF-1*α*, and VEGF in tumor tissues. Results are shown in Figure 4.

As shown in Figure 4, administration with different concentrations of TFAE significantly inhibited the expression of Bcl-2 in tumor tissues when compared with tissues belonging to negative control mice.

**Figure 5:**
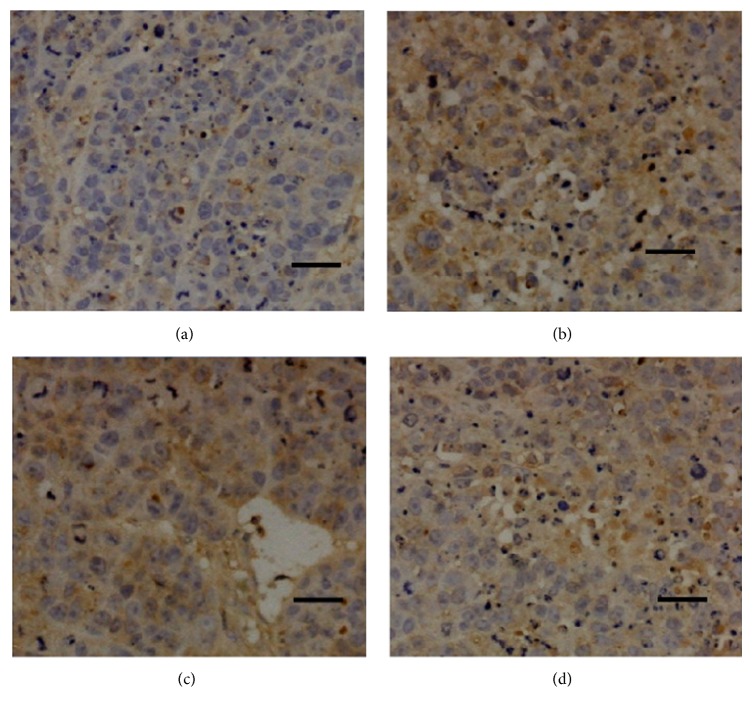
Expression of apoptosis-related protein Bax in tumor tissues (×100). (a) Negative control group; (b) TFAE low-dose group; (c) TFAE high-dose group; (d) positive control group.

As shown in Figure 5, TFAE at different concentrations effectively regulated the expression of Bax in tumor tissues compared with tissues of negative control mice. These findings well indicated that treatment with TFAE might induce the apoptosis in the tumor tissues.

**Figure 6:**
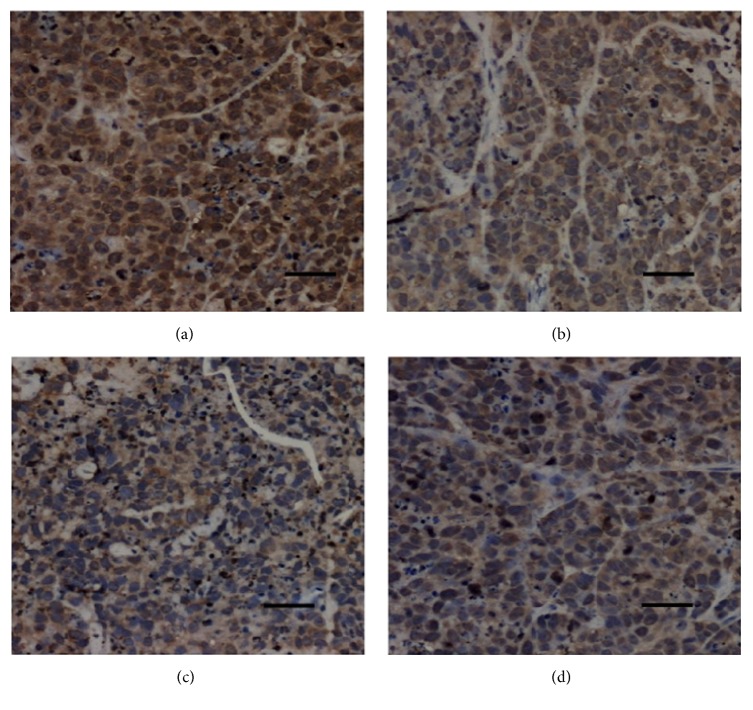
Expression of apoptosis-related protein HIF-1*α* in tumor tissues (×100). (a) Negative control group; (b) TFAE low-dose group; (c) TFAE high-dose group; (d) positive control group.

The protein HIF-1*α* is expressed in the cytoplasm or nucleus. The HIF-1*α* high-expressed cells show brown particles in nucleus and cytoplasm, while the HIF-1*α* low-expressed cells appear to be blue in cytoplasm. As shown in Figure 6, the expression level of HIF-1*α* in tissues of TFAE treated groups remarkably decreased compared with mice in negative control group, indicating that TFAE inhibited HIF-1*α* expression level in hepatoma cells, leading to the decreased ability to tolerate hypoxia and proliferation.

**Figure 7:**
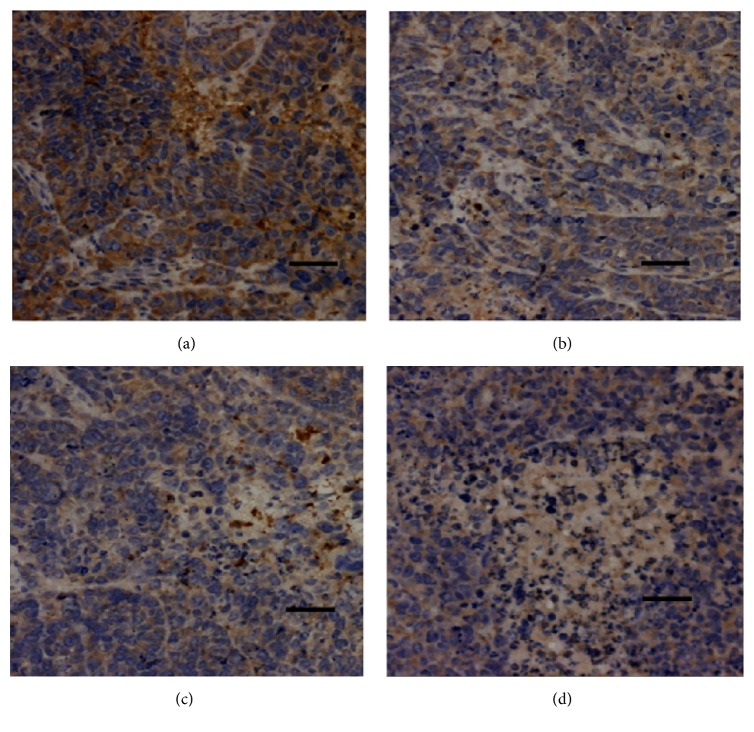
Expression of apoptosis-related protein VEGF in tumor tissues (×100). (a) Negative control group; (b) TFAE low-dose group; (c) TFAE high-dose group; (d) positive control group.

As shown in Figure 7, the expression of VEGF effectively decreased in the tissues of mice of TFAE high-dose group when compared with that of negative control group, suggesting that TFAE was able to inhibit the expression of VEGF in the tumor tissue, which resulted in the apoptosis of the cancer cells.

### 3.6. Expression Assay for Apoptosis-Related Proteins by Western Blot

**Figure 8:**
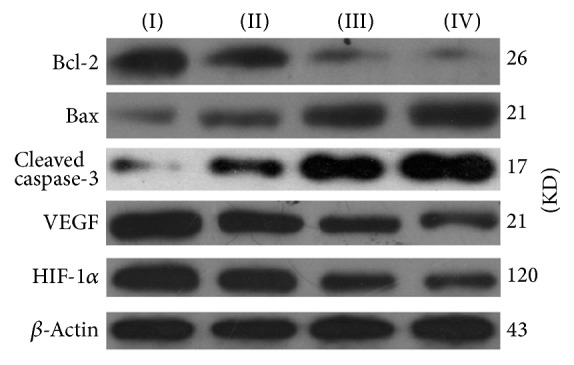
Expression assay for apoptosis-related proteins by Western blot. (I) Negative control group; (II) TFAE low-dose group; (III) TFAE high-dose group; (IV) positive control group.

**Figure 9:**
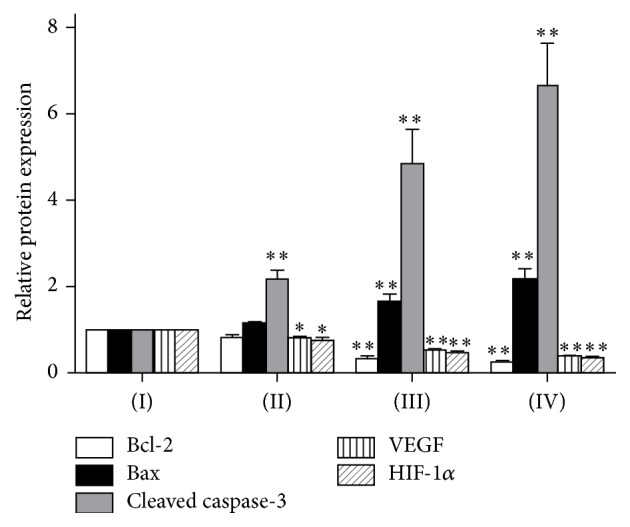
Relative protein expression assay for apoptosis-related proteins by Western blot. Values were presented as mean ± SD, ^*∗*^*p* < 0.05 and ^*∗∗*^*p* < 0.01 compared with negative control group.

The results are shown in Figures 8 and 9. Western blot assays showed that, when compared with mice of negative control group, the expression level of proapoptotic protein Bax in the mice significantly increased after the treatment with TFAE; meanwhile the expression of antiapoptotic protein Bcl-2 reduced remarkably in the tumor tissue, resulting in a higher ratio of Bax/Bcl-2. Furthermore, expression level of cleaved caspase-3 protein was effectively upregulated. The reducing expression of HIF-1*α* and VEGF was also shown in the Western blot assay.

## 4. Discussion

The current study investigated the potent antitumor activity of TFAE using a heterotopic liver cancer xenograft model* in vivo*. By detecting the changes of body weights and the tumor growth of the nude mice as well as determining the corresponding biochemical indicators involving blood system and functions of liver and kidney, it was confirmed that TFAE effectively inhibited tumor growth in the liver cancer ectopic xenograft models and, furthermore, had no significant effect on body weights, blood system, and functions of liver and kidney.

In order to further elucidate the mechanisms of the TFAE antitumor effects, immunohistochemical assay and Western blot assay were employed to explore the expression of proliferation-related proteins. Results showed that the expression level of proapoptotic protein Bax in tumor tissue was significantly elevated while expression of antiapoptotic Bcl-2 protein was effectively reduced, resulting in an increased Bax/Bcl-2 ratio. In addition, expression level of cleaved caspase-3 protein was increased and the reduced levels of HIF-1*α* and VEGF expressions were observed after the treatment of TFAE, suggesting the antitumor effect of TFAE by trigging apoptosis and inhibition of tumor angiogenesis.

Apoptosis is a process of programmed cell death, controlled by multiple signaling pathways, including the proteins of Bcl-2 family [12, 13] and caspase proteases [14, 15]. It is believed that the ratio of Bax/Bcl-2 activity level is a critical determinant of cell susceptibility to apoptosis, rather than the levels of individual proteins [16]. Increased Bax/Bcl-2 ratio and caspase-3 protein expression level induced by TFAE in this study are fully consistent with our previous study on HepG2 cells, confirming that the antitumor effect of TFAE is related to apoptosis* in vivo* [11].

Moreover, we also investigated the expression of HIF-1*α* protein in tumor tissue of nude mice with a liver cancer xenograft model. HIF-1*α* is a hypoxia-inducible factor-1*α* (hypoxia-inducible factor-1*α*), consisting of the heterodimer of HIF-1 protein with HIF-1*β*. The expression of HIF-1 was closely associated with the hepatocellular proliferation under hypoxic conditions. The physiological activity of HIF-1 mainly depends on the activity of HIF-1*α* subunit. Hypoxia is one of the key characteristics of the solid tumor microenvironment [17, 18], which leads to a series of changes in gene expression [19, 20]. Thus, hypoxia is closely related to tumor occurrence and development. Besides, the hypoxic environment can cause the tolerance of tumor cells to chemotherapy and radiation [21] and accelerate the tumor invasion and metastasis [22]. HIF-1 also plays a regulatory role in tumor cell apoptosis. Antiapoptosis genes of Bcl-2 family and survivin interact with the proapoptosis genes of Fas and p53 to regulate the apoptosis of liver cancer cells. Previous study indicated that HIF-1 possesses the control over expressions of Bcl-2, Bax, and survivin [23]. Expression of HIF-1*α* in tumor tissues was inhibited remarkably after the administration of TFAE, suggesting that the regulatory effect of TFAE on apoptosis of tumor cells is associated with the downregulated level of HIF-1*α*. However, the relationships between the expression of HIF-1*α* and the expression of Bcl-2 or between the expression of Bax and the expression of caspase need to be further confirmed by future studies.

HIF-1*α* has been proved to promote the expression of vascular endothelial growth factor (VEGF) [24]. VEGF is an important tumor vascular endothelial growth factor which can specifically induce the growth and proliferation of endothelial cells and accelerate angiogenesis occurrence and tumor growth [25, 26]. Additionally, VEGF inhibits tumor cell apoptosis [27]. Our results showed that TFAE could significantly reduce the expression level of VEGF in tumor tissue of nude mice and exert obvious effect on the inhibition of proliferation of tumor cells.

## 5. Conclusion

In this study, a heterotopic liver cancer xenograft model was adopted in nude mice to evaluate the anti-liver cancer effects of TFAE* in vivo* and explore the possible mechanisms. The heterotopic cancer xenograft model in nude mice has provided a convenient approach to be conducted and observed and, moreover, has short incubation period and high rate of tumor occurrence. It would greatly shorten the experimental period and would be suitable for screening of anticancer drugs.

In future studies, an orthotopic HCC animal model will be needed to make the results more accurate, reliable, and convincing. In addition, other animal models of liver cancer will be used to clarify the antihepatoma effect of TFAE* in vivo*.

## Figures and Tables

**Table 1:** The administration groups of nude mice.

Groups	Administration
Negative control	0.5% CMC-Na solution
TFAE high-dose	500 mg/kg TFAE
TFAE low-dose	125 mg/kg TFAE
Positive control	10 mg/kg 5-FU

**Table 2:** Changes in body weight of animal in different groups.

Groups	*n*	Body weight (g)	
		Before the experiment	After the experiment
Negative control	6	18.44 ± 2.56	24.63 ± 3.37
TFAE low-dose	6	18.56 ± 1.89	23.78 ± 3.01
TFAE high-dose	6	19.17 ± 2.03	24.12 ± 2.56
Positive control	6	18.75 ± 1.77	20.35 ± 2.86

**Table 3:** Assays about the blood system and functions of liver and kidney of the HCC animal model (*X* ± SD, *n* = 6).

Groups	Negative control group	TFAE low-dose group	TFAE high-dose group	Positive control group
RBC (×10^12^/l)	9.60 ± 1.62	9.54 ± 1.75	9.18 ± 1.34	9.12 ± 1.31
WBC (×10^9^/l)	12.26 ± 2.43	12.14 ± 2.39	11.66 ± 1.34	7.56 ± 1.44^*∗*^
PLT (×10^9^/l)	686.20 ± 105.99	678.02 ± 128.52	669.42 ± 95.46	618.78 ± 93.81
HB (g/l)	163.18 ± 24.01	165.60 ± 15.92	158.49 ± 27.73	154.74 ± 26.53
ALT (U/l)	73.73 ± 17.36	80.27 ± 24.72	81.57 ± 31.28	168.37 ± 42.83^*∗*^
AST (U/l)	377.27 ± 65.37	407.82 ± 88.31	383.60 ± 80.16	702.63 ± 106.45^*∗*^
BUN (mmol/l)	12.56 ± 2.38	13.71 ± 2.74	13.18 ± 1.77	16.84 ± 3.76^*∗*^
Cr (*µ*mol/l)	12.30 ± 3.01	12.04 ± 2.06	13.17 ± 2.65	15.45 ± 3.54

^*∗*^ *p* < 0.05, compared with negative control.
